# Diagnosis of Asymptomatic Biliary Ascariasis by Abdominal Ultrasound in a Non-Endemic Area

**DOI:** 10.7759/cureus.33599

**Published:** 2023-01-10

**Authors:** Carolina Silva, Inês C Gonçalves, Sara Neves, Diana C Ferreira, Sofia R Valdoleiros

**Affiliations:** 1 Infectious Diseases, Centro Hospitalar Universitário do Porto, Porto, PRT; 2 Family Medicine, Unidade de Cuidados de Saúde Personalizados Fragoso, Barcelos, PRT; 3 Infectious Diseases, Centro Hospitalar Universitário de São João, Porto, PRT; 4 Infectious Diseases, Faculty of Medicine of the University of Porto, Porto, PRT

**Keywords:** diagnostic imaging, gallbladder, asymptomatic infections, ascariasis, parasitology

## Abstract

Biliary ascariasis is rare in non-endemic areas. This infection is associated with severe complications of the biliary tract, which can become a medical emergency. Treatment with oral anthelmintics is often effective, but, in some cases, surgery is required.

We describe an unusual case of ultrasound diagnosis of biliary ascariasis in the gallbladder in a patient who, besides residing in a low-prevalence area of the infection, did not present with biliary tract manifestations. We intend to raise awareness of this clinical entity in non-endemic areas, where this diagnosis is not usually considered. A brief review of the subject is also presented.

## Introduction

Ascariasis is caused by *Ascaris* species, mainly *Ascaris lumbricoides* [1]. It is the most common helminthic infection worldwide [2-4], affecting at least 25 to 33% of the world population [3,5,6]. It is estimated that more than one billion people are infected [4].

Ascariasis is endemic in low-income tropical and subtropical countries [2]. In high-income areas, the prevalence of the infection decreased significantly after the introduction of modern sanitation in the early 20thcentury, but it can occur among travelers to high-prevalence areas [2,5,6]. Recent studies about ascariasis in Mediterranean countries are lacking, but the incidence of intestinal parasitosis is usually low [2,5]. 

Transmission occurs primarily via ingestion of water or food contaminated with *Ascaris* eggs [1]. Most patients have asymptomatic infection [3]. Symptoms may occur in an early phase of the infection, during the larval migration stage, as pulmonary manifestations [5]. The symptomatic intestinal disease occurs in the adult worm stage, which can be associated with multiple complications, such as intestinal obstruction or hepatobiliary and pancreatic manifestations [5].

Biliary ascariasis is very uncommon in non-endemic areas [1,2]. It has a female preponderance (female-to-male ratio of 3:1) and is commonly seen in the mid-thirties [5]. It occurs when the parasite, dwelling in the small intestine, inadvertently migrates into the bile ducts [1]. Biliary colic, acute cholangitis, acute cholecystitis, pancreatitis, and hepatic abscess are possible manifestations [3]. Nonetheless, the asymptomatic presence of the parasite in the biliary tract has not been studied in detail, considering that the execution of abdominal imaging in the absence of clinical manifestations is not a routine medical practice in most scenarios [5].

Most cases of biliary ascariasis resolve after conservative medical treatment with an oral anthelmintic [1,3]. Surgery may be required when treatment fails to eradicate the infection [3].

## Case presentation

An 86-year-old man with a past medical history of prostate cancer in remission after radiotherapy, arterial hypertension, and chronic pulmonary obstructive disease presented to the primary care health system for his annual medical evaluation. The patient did not have any complaints, such as fever, weight loss, nausea, vomiting, diarrhea, abdominal pain, jaundice, or pruritus. He did not notice any worms in his stools, nor any aggravated respiratory symptoms. Physical examination was normal.

He previously worked as a farmer and was living in a rural area of Portugal, where he consumed well water. He only traveled outside of Europe to India in 1955 for military duty, where he stayed for two years. He was never diagnosed or received any treatment for a parasitic infection.

The complete blood count showed isolated eosinophilia of 0,52 x10^3^/μL; the hepatic function panel and liver enzymes were not altered.

**Figure 1:**
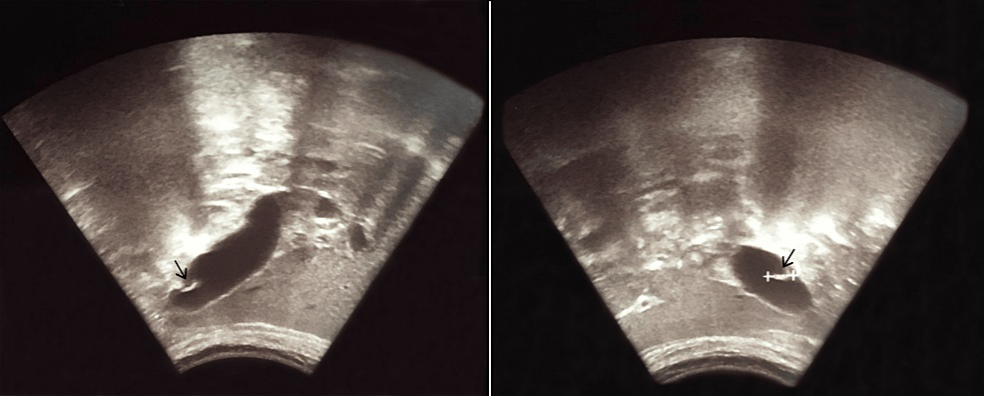
Abdominal ultrasound showing thickening of the gallbladder wall, with visualization of hyperechoic structures inside it, creating a tubular coiled appearance (arrow), without acoustic shadowing.

A routine abdominal ultrasound was performed, which revealed normal liver parenchyma, without focal lesions, with discrete dilation of the intrahepatic bile ducts. There was thickening of the gallbladder wall, with visualization of hyperechoic structures inside it with hypoechoic center lines like a rail, creating a tubular coiled appearance, without acoustic shadowing, and with apparent writhing movements (Figure 1). The main bile duct was normal. These imaging findings were compatible with biliary ascariasis. A chest radiograph was performed, and it did not show any significant alterations.

He was treated with 400 mg of oral albendazole in a single dose. Stool examination was only possible after treatment. No adult worms or eggs were visualized, on direct microscopy and following concentration techniques.

A complete resolution of the eosinophilia on the blood count was observed. A new abdominal ultrasound did not show any endoluminal content in the gallbladder.

## Discussion

Ascariasis is a common helminthic infection in rural areas of low-income countries [1]. Individuals get infected primarily by fecal-oral transmission [1]. Most cases of ascariasis are asymptomatic [3]. Nonetheless, given the high prevalence of the infection, the burden of symptomatic disease is relatively high [2]. In the early phase of the infection, which occurs four to 16 days following egg ingestion, the migration of the worm through the lungs may be associated with transient respiratory symptoms and eosinophilic pneumonitis [6,7].

The late phase of infection (six to eight weeks after egg ingestion) is characterized by nonspecific symptoms, such as abdominal pain, anorexia, nausea, vomiting, and diarrhea [6]. Complications of this phase of ascariasis include intestinal obstruction, malnutrition, hepatobiliary involvement, and pancreatitis [5]. Macroscopic adult worms are passed in the stool [6]. Peripheral eosinophilia may be observed but is more frequent during the early phase [1].

Biliary ascariasis is an important cause of common bile duct obstruction and stricture in high-endemic areas [5]. It can manifest in a multitude of entities, most commonly biliary colic, acute cholangitis, and acute cholecystitis [3,5]. Thus, it can become a severe medical condition, sometimes requiring emergency surgery [5].

In the presented case, the fact that it is an asymptomatic infection hinders the possibility of determining the date of transmission. Even though adult worms have a lifespan of 10 to 24 months in the stools [1], some cases of biliary ascariasis can present as chronic worm infestation [1]. Nevertheless, these cases are usually symptomatic with recurrent biliary colic [1]. Consequently, in this case, it is not possible to conclude if the infection was acquired in his country of residence (which is considered a low-prevalence area), or around the time he traveled overseas to India (an endemic area), even though that occurred more than 50 years ago. Persistent infection occurs through frequent re-exposure and reinfection of the host [1].

The diagnosis of biliary ascariasis is based on anamnesis and physical examination, as well as stool microscopy for eggs or via examination of adult worms; in some cases, the eggs or worms can be seen in the bile [1,5]. In the absence of isolation of the microorganism, typical imaging findings can be helpful [5,8]. Ultrasonography is an excellent modality to visualize *Ascaris* in the stomach, duodenum, biliary tree, and pancreatic ducts [3,5,9]. In the case of biliary ascariasis, ultrasound findings include a long, coiled echogenic structure, without posterior shadowing, an echogenic strip with a central anechoic line, a gallbladder with a septate appearance caused by an echogenic structure, associated with random movements of these structures [3,5,8,9]. The asymptomatic presence of the worm in the gallbladder is of undetermined significance, mainly because of the limited studies in this scenario, but theoretically, it may precede the development of complications.

All patients with ascariasis need anthelmintic treatment, even those with asymptomatic infection [1,3]. Several anthelmintic drugs such as pyrantel pamoate, mebendazole, ivermectin, and levamisole have been used to effectively treat ascariasis, but a single dose of 400 mg of albendazole is often the agent of choice [3]. In biliary ascariasis cases, if conservative medical treatment fails to eradicate the infection, endoscopic retrograde cholangiopancreatography and surgery are the treatments of choice [1,3]. Nevertheless, mortality is low, and the prognosis is good, especially if a correct and timely diagnosis is made [5].

Due to the very typical findings in the ultrasound, it was decided to treat the presented patient with an anthelmintic drug. Microbiological confirmation of the diagnosis was not possible; in fact, in the absence of intestinal symptoms and after the administration of treatment, the sensitivity of stool microscopy is very low. However, the resolution of the eosinophilia and the disappearance of the imaging findings after treatment support the diagnosis. The treatment may have prevented the development of symptomatic complications of biliary ascariasis.

## Conclusions

Biliary ascariasis is a treatable disease. However, it can be difficult to diagnose even in the presence of symptoms; a high index of suspicion is required for the diagnosis. Typical ultrasound findings of the infection should be recognizable even in non-endemic areas, so that the clinicians can properly approach the patients and prescribe adequate treatment.
